# Comparing the hemodynamic effects of ketamine versus fentanyl bolus in patients with septic shock: a randomized controlled trial

**DOI:** 10.1007/s00540-024-03383-9

**Published:** 2024-08-18

**Authors:** Maha Mostafa, Ahmed Hasanin, Basant Reda, Mohamed Elsayad, Marwa Zayed, Mohamed E. Abdelfatah

**Affiliations:** Department of Anesthesia and Critical Care Medicine, Faculty of Medicine, Cairo University, Cairo, Egypt

**Keywords:** Septic shock, Mechanical ventilation, Sedation, Ketamine, Fentanyl, Cardiac output, Hypotension

## Abstract

**Background:**

Ketamine and fentanyl are commonly used for sedation and induction of anesthesia in critically ill patients. This study aimed to compare the hemodynamic effects of ketamine versus fentanyl bolus in patients with septic shock.

**Methods:**

This randomized controlled trial included mechanically ventilated adults with septic shock receiving sedation. Patients were randomized to receive either 1 mg/kg ketamine bolus or 1 mcg/kg fentanyl bolus. Cardiac output (CO), stroke volume (SV), heart rate (HR), and mean arterial pressure (MAP) were measured at the baseline, 3, 6, 10, and 15 min after the intervention. Delta CO was calculated as the change in CO at each time point in relation to baseline measurement. The primary outcome was delta CO 6 min after administration of the study drug. Other outcomes included CO, SV, HR, and MAP.

**Results:**

Eighty-six patients were analyzed. The median (quartiles) delta CO 6 min after drug injection was 71(37, 116)% in the ketamine group versus − 31(− 43, − 12)% in the fentanyl group, *P *value < 0.001. The CO, SV, HR, and MAP increased in the ketamine group and decreased in the fentanyl group in relation to the baseline reading; and all were higher in the ketamine group than the fentanyl group.

**Conclusion:**

In patients with septic shock, ketamine bolus was associated with higher CO and SV compared to fentanyl bolus.

**Clinical trial registration:**

Date of registration: 24/07/2023. ClinicalTrials.gov Identifier: NCT05957302. URL: https://clinicaltrials.gov/study/NCT05957302.

**Supplementary Information:**

The online version contains supplementary material available at 10.1007/s00540-024-03383-9.

## Introduction

A considerable proportion of patients with circulatory shock requires sedation or anesthesia and most of intravenous anesthetic agents, such as propofol and fentanyl, have a negative hemodynamic profile which makes the intervention a challenging hot topic in anesthetic and intensive care practice [1, 2]. Cardiac output is the primary determinant of global oxygen delivery to organs as well as the oxygen content [3]‚ and maintaining stable cardiac output in critically ill patients is of most importance to avoid further organ damage [4]. Patients with septic shock are characterized by diminished systemic vascular resistance which increases the role of the cardiac output in oxygen delivery; therefore, maintenance of the cardiac output during sedation and anesthesia is crucial. Fentanyl is commonly used in induction of anesthesia and/or sedation in critical care units [5]. However, it is unclear whether its use, especially as bolus, is the safest in patients with shock or not. Several data in other patient groups suggest that opioids are responsible, or at least contributors, in hypotension in several clinical contexts [6, 7]. Ketamine is another commonly used drug for sedation and induction of anesthesia [8, 9] and is characterized by cardiovascular stimulatory effect due to release of endogenous catecholamines [10]. However, laboratory data on the isolated human myofibers suggest that ketamine had a direct myocardial depressive effect [11]; accordingly, many experts believe that ketamine might have a negative hemodynamic effect in catecholamine-depleted patients such as patients with septic shock. Clinical data showed contradictory results for the effects of ketamine on the hemodynamic profile in critically ill patients [12–16]. Therefore, this study was designed to evaluate the effect of ketamine versus fentanyl bolus on cardiac output in patients with septic shock.

## Patients and methods

This randomized controlled trial was conducted in the surgical intensive care unit at Cairo University Hospital between August and December 2023. This study was approved by the University’s Research Ethics Committee (MS-47-2023) and written informed consent was obtained from the subject’s guardian before participating in the trial. The trial was registered prior to patient enrollment at clinicaltrials.gov (NCT05957302).

We included adult (> 18 years) patients with septic shock on vasopressor therapy to maintain mean arterial pressure > 65 mmHg despite adequate fluid therapy and serum lactate level > 2 mmol/L [17]. All the included patients were mechanically ventilated who needed a bolus of sedative to resume sedation after a period of sedation vacation. We enrolled the patients after the resuscitative phase of septic shock management (after shock recognition, achieving euvolemia [18] and reaching a stable dose of vasopressor to achieve mean arterial pressure > 65 mmHg). Patients were evaluated for fluid responsiveness and hypovolemia was corrected according to the surviving sepsis campaign guidelines. The main methods for evaluation of volume responsiveness were passive leg raising test, fluid challenge test or IVC variability and the choice of the test was left according to the nature of the case and discretion of the attending intensivist [19].

Exclusion criteria included hemodynamic instability (mean arterial pressure < 65 mmHg) despite appropriate volume replacement and vasopressor therapy, noradrenaline infusion rate < 0.05 mcg/kg/min, poor cardiac window on the ultrasound, known allergy to any of the study’s drugs, increased intracranial tension and pregnancy.

Patients were randomized using computer generated random numbers at a 1:1 ratio. Group assignment according to the randomization number in addition to the drug preparation instructions were enclosed in a consequentially numbered opaque envelope. An independent research assistant handled the envelope-opening and drug preparation with no further involvement in the study.

### Drug preparation

Ketamine group: 100 mg of ketamine was diluted in 10 mL normal saline (10 mg /mL) and patient received 0.1 mL/kg (1 mg/kg).

Fentanyl group: 100 mcg of fentanyl was diluted in 10 mL normal saline (10 mcg/mL) and patient received 0.1 mL/kg (1 mcg/kg).

According to our local protocols, all patients received a period of sedation vacation, achieved by interruption of the ongoing sedation infusion and the Richmond Agitation Sedation Scale (RASS) was assessed periodically for wakefulness (defined as RASS ≥ -1 with the ability to obey simple command). When resumption of sedation was decided, according to the attending intensivist discretion, the patients received a bolus of the study drug according to randomization and the target level of RASS was − 2 to 0 [20]. All patients were monitored by a 5-lead electrocardiogram, pulse oximetry, and non-invasive blood pressure monitor.

Hypotension was defined as mean arterial pressure < 65 mmHg and was managed by increasing the norepinephrine infusion rate by 50%.

Bedside echocardiography was performed by a blinded-experienced intensivist. The left ventricular outflow diameter (LVOT) was measured at the parasternal long-axis view. Then the velocity time integral (VTI) was measured at the apical five-chamber view using pulsed-wave Doppler. The average of three VTI readings was recorded.

The intensivist was blinded to the intervention and only collected data for the LVOT diameter and LVOT-VTI without any further involvement into the study. Stroke volume and cardiac output were calculated later by the data collector according to the following equations:

$${\text{Stroke volume }} = ( \pi \times {\text{ (LVOT diameter}}/{2)}^{2} \times {\text{ VTI}})$$

$${\text{Cardiac output}} = {\text{stroke volume}} \times {\text{ heart rate}}.$$

Delta cardiac output was calculated as the percentage of change in the cardiac output at each time point in relation to the baseline measurement.

In patients with atrial fibrillation, the VTI assessment was possible if the RR interval ratio of the two-preceding beats was close to 1 [21].

The hemodynamic variables, namely cardiac output, stroke volume, heart rate, and mean arterial pressure as well as the RASS, were recorded before drug administration and at 3, 6, 10, and 15 min after drug administration.

### Primary outcome

Delta cardiac output 6 min after drug administration.

### Secondary outcomes

LVOT-VTI, cardiac output, stroke volume, heart rate, and mean arterial pressure, incidence of post-induction hypotension. Acute Physiology and Chronic Health Evaluation (APACHE) II score, Sequential Organ Failure Assessment (SOFA) score, days of mechanical ventilation and norepinephrine infusion rate were recorded at the time of enrollment. Patient’s demographic data, comorbidities, source of sepsis, and type of sedative drug before the sedation vacation were also recorded.

### Sample size

In a pilot study on 7 mechanically ventilated patients with septic shock, the delta cardiac output 6 min after fentanyl bolus was − 23 ± 15%; therefore, a sample size of 72 would achieve a study power of 80% to detect a difference of 10% in delta cardiac output between the two groups assuming a common standard deviation of 15% at a significance level (alpha) of 0.05. Eighty-six patients (43 patients in each group) were included to compensate for possible dropouts. Sample size was calculated using MedCalc Statistical Software version 14.10.2 (MedCalc Software bvba, Ostend, Belgium).

### Statistical analysis

Statistical package for social science (SPSS) software, version 26 for Microsoft Windows (IBM Corp., NY, USA) was used for data analysis. Categorical data are presented as frequency (%) and were analyzed using the Chi squared test. Continuous data were checked for normality using the Shapiro–Wilk test and are presented as mean ± standard deviation or median (quartiles) as appropriate. Continuous data were compared using the Student’s t test if they were normally distributed or the Mann–Whitney test if they were skewed. Repeated measures were analyzed using the analysis of variance for repeated measures. The Bonferroni test was used for adjustment for multiple comparisons. A *P* value less than 0.05 was considered statistically significant.

## Results

**Fig. 1:**
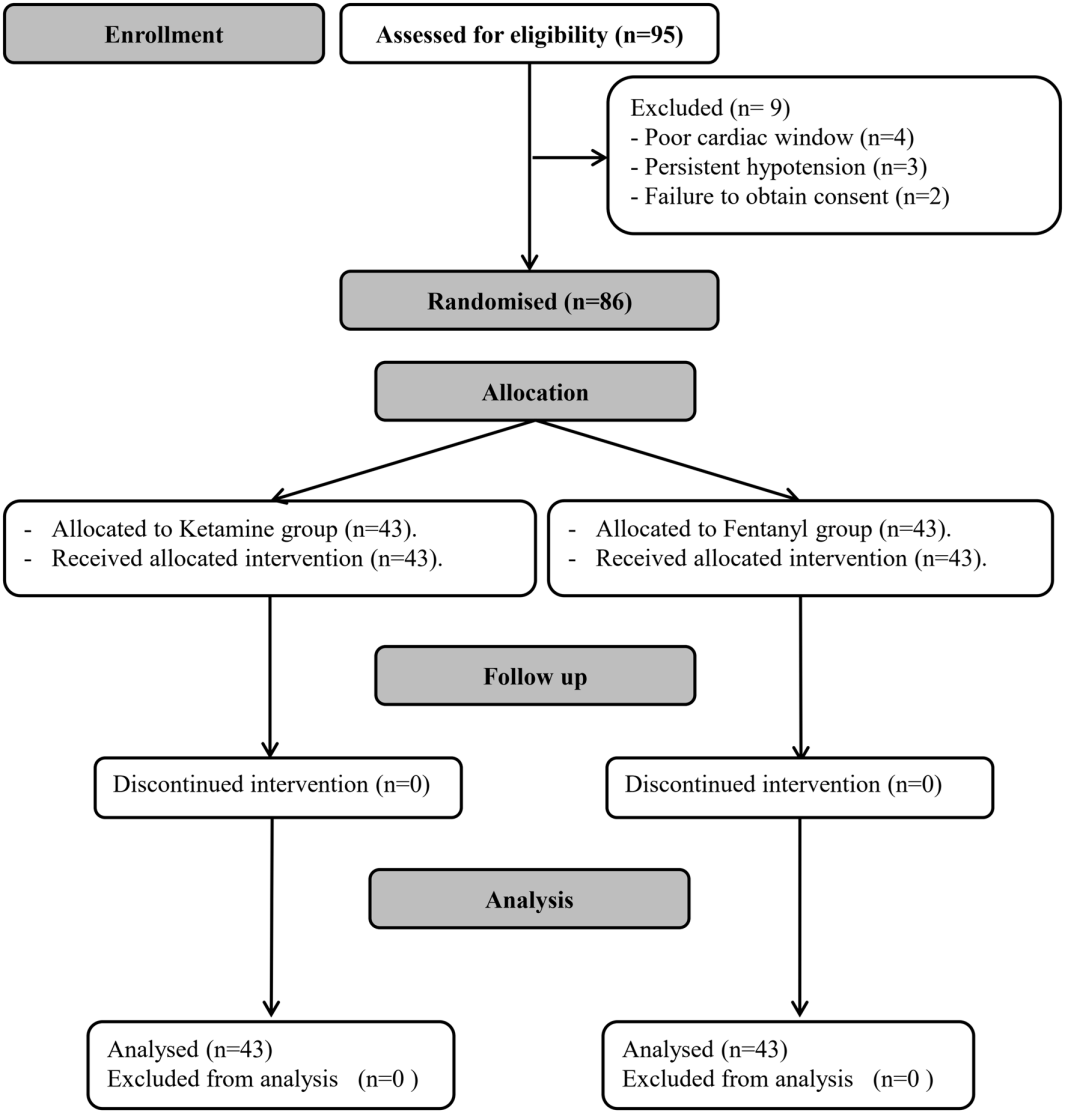
CONSORT’s flowchart

Ninety-five patients were evaluated for eligibility for the study. Nine patients were excluded for not meeting the inclusion criteria. Eighty-six patients were randomized to receive one of the two interventions and were available for the final analysis. (Fig. 1).

Demographic data and baseline characteristics were comparable between both groups. (Table 1).

**Table 1:** Demographic data and baseline characteristics

	Ketamine group (n = 43)	Fentanyl group (n = 43)	*P* value
Age (years)	60 (48, 69)	62 (55, 68)	0.554
Height (cm)	170 (160, 174)	165 (160, 170)	0.251
Weight (kg)	65 (60, 74)	65 (55, 70)	0.253
Male gender (%)	21 (55%)	17 (46%)	0.420
Comorbidity Hypertension Diabetes mellitus Atrial fibrillation Chronic kidney disease Cerebrovascular stroke Epilepsy Bronchial asthma	13 (30%) 19 (44%) 3 (7%) 1 (2%) 1 (2%) 1 (2%) 1 (2%)	15 (35%) 24 (56%) 2 (5%) 2 (5%) 0 (0%) 0 (0%) 0 (0%)	0.645 0.281 0.949 1.000 1.000 1.000 1.000
Source of sepsis Abdominal Soft tissue Respiratory	27 (63%) 15 (35%) 1 (2%)	25 (58%) 18 (42%) 0 (0%)	0.509
Sedation drug before the sedation vacation Fentanyl only Fentanyl and propofol Fentanyl and midazolam	39 (91%) 3 (7%) 1 (2%)	42 (98%) 1 (2%) 0 (0%)	0.348
Fentanyl infusion rate before sedation vacation (mcg/h)	50 (50, 50)	50 (50, 50)	0.293
Norepinephrine rate (mcg/kg/min)	0.3 (0.2, 0.4)	0.3 (0.2, 0.4)	0.626
SOFA score	4 (3, 6)	4 (3, 5)	0.881
APACHE II score	10 (8, 13)	10 (8, 12)	0.906
Days of mechanical ventilation	2 (1, 2)	2 (1, 2)	0.484
Mean arterial pressure (mmHg)	88 ± 10	87 ± 11	0.849
Heart rate (beat/min)	97 ± 16	94 ± 21	0.410
Stroke volume (mL)	67 ± 42	75 ± 29	0.130
Cardiac output (L/min)	6.3 ± 3.8	7.0 ± 3.2	0.160

Data are presented as mean ± standard deviation, median (quartiles), and frequency (%)

*APACHE* Acute Physiology and Chronic Health Evaluation, *SOFA* Sequential Organ Failure Assessment

The delta cardiac output was positive in the ketamine group and negative in the fentanyl group and it was significantly different between the two groups at all timepoints after drug injection (Table 2). The incidence of post-induction hypotension was not significantly different between the two groups (Table 2).

**Table 2:** Hemodynamic outcomes

	Ketamine group (*n* = 43)	Fentanyl group (*n* = 43)	*P* value
Delta cardiac output (%) 3 min 6 min 10 min 15 min	30 (6, 44) 71 (37, 116) 49 (20, 98) 40 (19, 70)	− 20 (− 34, − 6) − 31 (− 43, − 12) − 25 (− 41, − 10) − 9 (− 33, 11)	< 0.001 < 0.001 < 0.001 < 0.001
Post-induction hypotension	0 (0%)	2 (5%)	0.494

Data are presented as median (quartiles) and frequency (%)

**Fig. 2:**
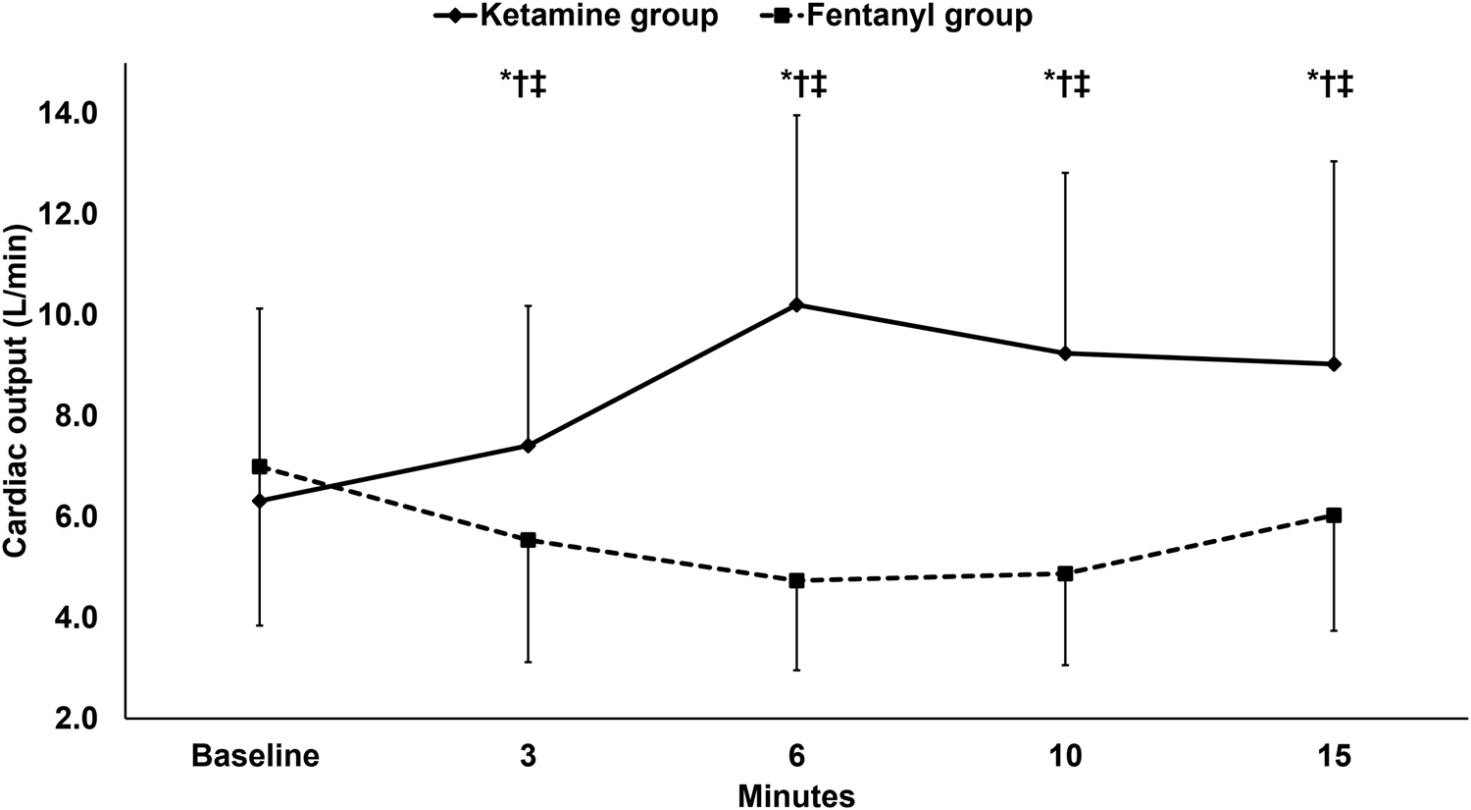
Cardiac output. Markers are means and error bars are standard deviations. * denotes statistical significance between both groups, † denotes statistical significance compared to the baseline reading within the ketamine group, ‡ denotes statistical significance compared to the baseline reading within the fentanyl group

**Fig. 3:**
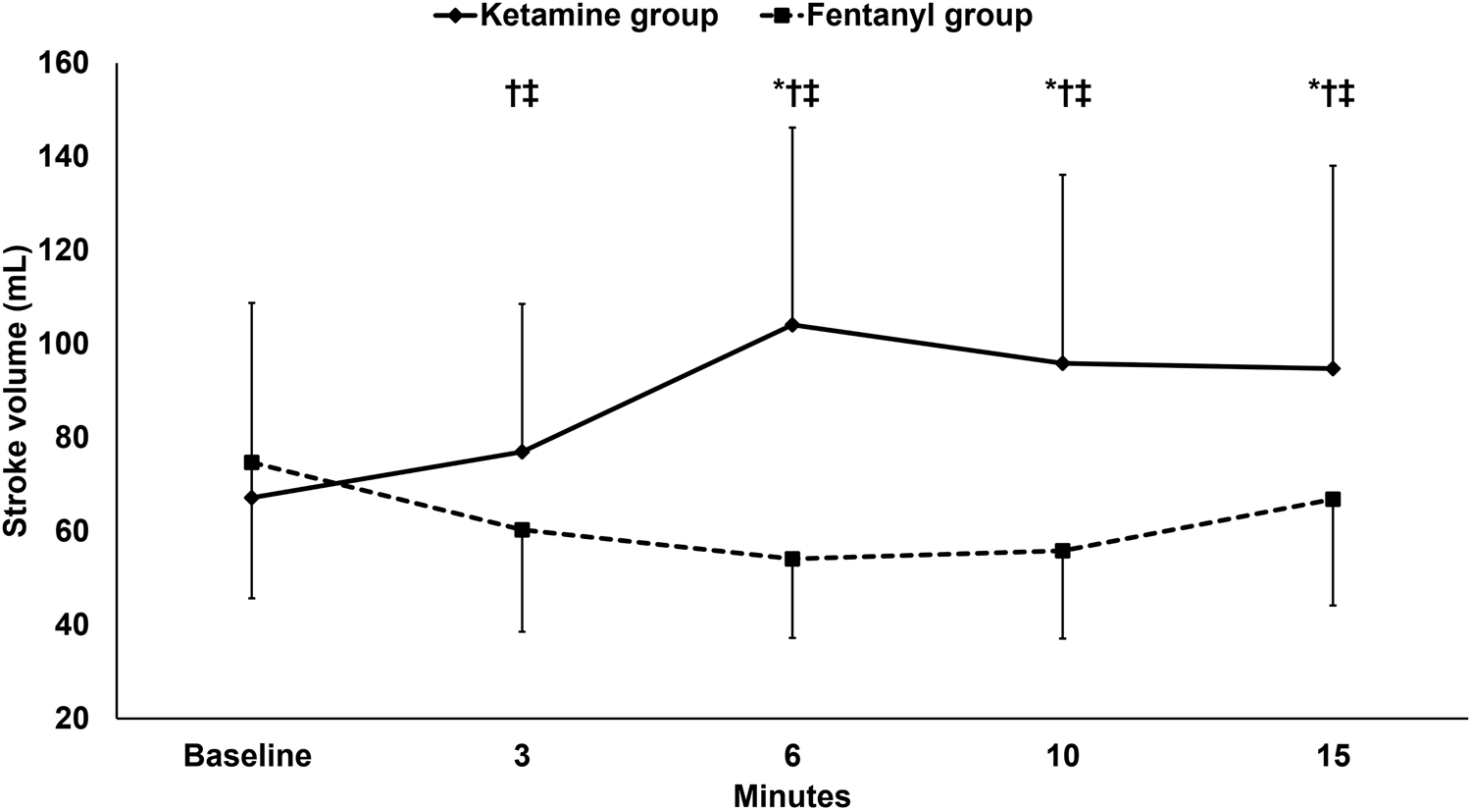
Stroke volume. Markers are means and error bars are standard deviations. * denotes statistical significance between both groups, † denotes statistical significance compared to the baseline reading within the ketamine group, ‡ denotes statistical significance compared to the baseline reading within the fentanyl group

The hemodynamic parameters, namely LVOT-VTI, (supplementary Fig. 1), cardiac output, stroke volume (Figs. 2 and 3), mean arterial pressure, and heart rate (supplementary Fig. 2 and 3) increased in the ketamine group and decreased in the fentanyl group in relation to the baseline reading; and all were higher in the ketamine group than in the fentanyl group.

**Fig. 4:**
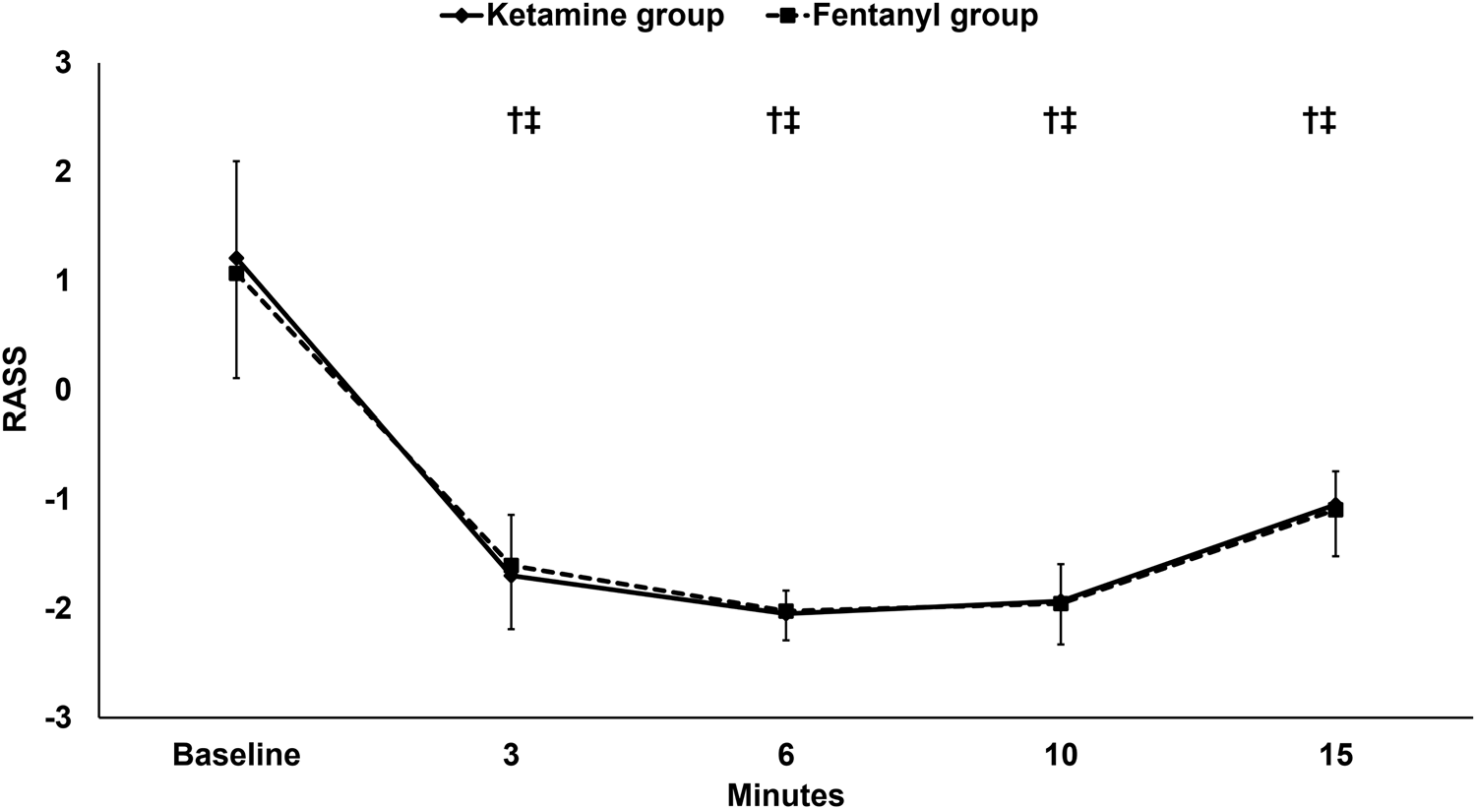
RASS. Markers are means and error bars are standard deviations. † denotes statistical significance compared to the baseline reading within the ketamine group, ‡ denotes statistical significance compared to the baseline reading within the fentanyl group. *RASS* Richmond Agitation Sedation Scale

The RASS decreased in both groups after drug administration, and none of the patients needed additional sedation bolus. Furthermore, the RASS was similar between the two groups at all timepoints (Fig. 4).

## Discussion

We report that a bolus of ketamine produced better hemodynamic profile compared to fentanyl bolus in patients with septic shock. In our patients, ketamine increased the cardiac output by 71% while fentanyl decreased the cardiac output by 31%. Our results revealed that the higher cardiac output in the ketamine group is not only due to the higher heart rate, but also due to higher stroke volume. On the other hand, fentanyl decreased the stroke volume denoting a cardiac depressant effect. To the best of our knowledge, this is the first randomized controlled trial which compared the effect of the two drugs at induction bolus doses on the cardiac output in patients with septic shock receiving norepinephrine.

The effect of different induction hypnotic agents on the hemodynamic profile is well-studied in patients with stable arterial blood pressure. Ketamine is a drug with known sympathomimetic activity that increases the cardiac output by ≈40% [22–24]; thus, it has the most stable hemodynamic profile compared to other anesthetic drugs [25]. Whether the positive cardiovascular effect of ketamine is due to increased heart rate only or due to increase both heart rate and cardiac systolic function is an unresolved point. Some in vitro studies reported that ketamine impairs cardiac contractility in failing myocardium [26, 27], supporting the assumption that ketamine only increases the heart rate and systemic vascular resistance and not the cardiac systolic function. Our results showed increased stroke volume after ketamine bolus debating the assumption of the previous in vitro reports and supporting the safety of the drug in failing heart. No clinical reports had evaluated the effects of ketamine on the cardiac output in patients with shock. However, some studies compared ketamine to several hypnotic drugs, such as propofol, midazolam, and etomidate, in emergency endotracheal intubation and found that ketamine was comparable to etomidate and superior to propofol and midazolam with regard to the hemodynamic profile [28, 29]. A small randomized controlled trial included 32 patients compared ketamine to fentanyl during rapid sequence induction of anesthesia and showed that ketamine was superior to fentanyl, and this is in line with the current study. However, the current study has several strengths, such as the larger sample size (86 versus 32 patients), the use of more valid method for cardiac output measurement (echocardiography versus electrical cardiometry), the evaluation of the effect of the study drug without any confounders (neuromuscular blockers–endotracheal intubation), and the longer period of evaluation [30]. In the current study, the cardiac output was selected as the principal outcome for being the main determinant of oxygen delivery and tissue prefusion with the change at 6 min as the primary outcome. In the pilot study, the hemodynamic change reached its maximum value 6 min after injection of the drug bolus. Furthermore, we tried to select practical intervals for our measurements because long intervals would probably miss some effects and short intervals might not be feasible to perform accurate measurement.

Fentanyl is an opioid drug commonly used for anesthesia and sedation in several types of patients including those with cardiovascular pathologies [31]; however, its effects in hemodynamically unstable patients were not investigated in comparison to other anesthetic agents. Our results showed a negative hemodynamic response to fentanyl in patients with shock raising a safety concern for the use of fentanyl in hemodynamically vulnerable patients.

In this study, the two study drugs were administered at doses which are commonly used in daily practice. Ketamine bolus is usually given as 1–2 mg/kg while fentanyl bolus usually ranges at 50–100 mcg (≈1 mcg/kg) [20, 32]. furthermore, from the data of this study, both drugs produced similar sedative effect and no additional sedative boluses were needed; therefore, we believe that the doses used in this study are equipotent and produced an adequate sedation level.

Sedation is daily performed in every intensive care unit and is constantly needed in patients with profound circulatory failure who need invasive mechanical ventilation [33]. The current sedation guidelines recommend an analgesia-first and minimal sedation strategy with opioid drugs being at the top-frequent used drugs for analgosedation [34, 35]. Ketamine is also mentioned in the guidelines with uprising evidence about its opioid-sparing effect besides its favorable cardiovascular and respiratory effects [35]. There is lack of data in the current guidelines about the preference of one drug over the other in patients with hemodynamic instability as most of the hypnotic drugs aggravate the pre-existing hypotension. A recent meta-analysis showed that adding of ketamine to analgosedation regimens could decrease opioid consumption; however, this meta-analysis did not report sufficient data about the hemodynamic sequelae of ketamine in unstable patients [36].

Patients with septic shock usually need surgical intervention for source control. Maintaining blood pressure in these patients is at most importance to reduce mortality; however, these patients usually have septic induced cardiomyopathy, in addition, most anesthetic drugs have negative cardiovascular effects which are exaggerated by the positive pressure ventilation [37], and increased pulmonary vascular resistance [38]. Hypotension represents the commonest complication after induction of anesthesia in critically ill patients [39]; furthermore, the presence of pre-intubation shock increases the likelihood of peri-intubation cardiac arrest [40]. Two large randomized controlled trials (namely the PrePARE and PREPARE-II) showed that administration of fluid bolus did not prevent cardiovascular collapse during endotracheal intubation in critically ill patients [41, 42] and this supports the assumption that the main line for maintenance of stable blood pressure in these patients is through the proper selection of hypnotic drugs besides the use of vasopressors, when needed.

Our results provide high-quality evidence for the comparison of fentanyl and ketamine in a critical population. For being performed in humans, the results of our study challenge the previous data which reported a direct cardiac inhibitory effect in vitro [26, 27]. The adequate sample size; the randomized controlled double-blinded design; and the detailed hemodynamic evaluation strengthen our findings and support the use of ketamine in induction and maintenance of sedation in patients with septic shock. Another important point in our study is inclusion of patients with more severe shock than previous reports as all our participants were on norepinephrine infusion. We used the common doses for induction to have a pragmatic design which is applicable for both anesthesia and sedation in patients with circulatory shock [28].

The study has some limitations such as being performed in a single center; however, our hospital is a tertiary hospital which receives referred complicated cases from several smaller hospitals and this adds more generalizability to our findings. All patients in our study were in septic shock and the results might differ in other types of shock. We did not include extremes of age in the study; future research might be needed to confirm our findings in children and elderly patients.

In conclusion, in patients with septic shock, ketamine bolus produced higher cardiac output and stroke volume compared to fentanyl bolus. The higher stroke volume after receiving ketamine compared to fentanyl reflects that the former drug has a direct cardiac stimulant effect while the latter drug has a cardiac inhibitory action.

## Supplementary Information

Below is the link to the electronic supplementary material.

Supplementary file1 (DOCX 413 KB)

## Data Availability

The datasets used and/or analyzed during the current study are available from the corresponding author upon reasonable request.
