# Supplementary material for: Comparing the hemodynamic effects of ketamine versus fentanyl bolus in patients with septic shock: a randomized controlled trial
Source: J Anesth. 2024 Aug 18;38(6):756–64. doi: 10.1007/s00540-024-03383-9 (PMC11584442; doi:10.1007/s00540-024-03383-9)
Supplement: Supplementary file 1 — Supplementary file1 (DOCX 413 KB) [file 540_2024_3383_MOESM1_ESM.docx]

**Comparing the hemodynamic effects of ketamine versus fentanyl bolus in patients with septic shock: a randomized controlled trial**


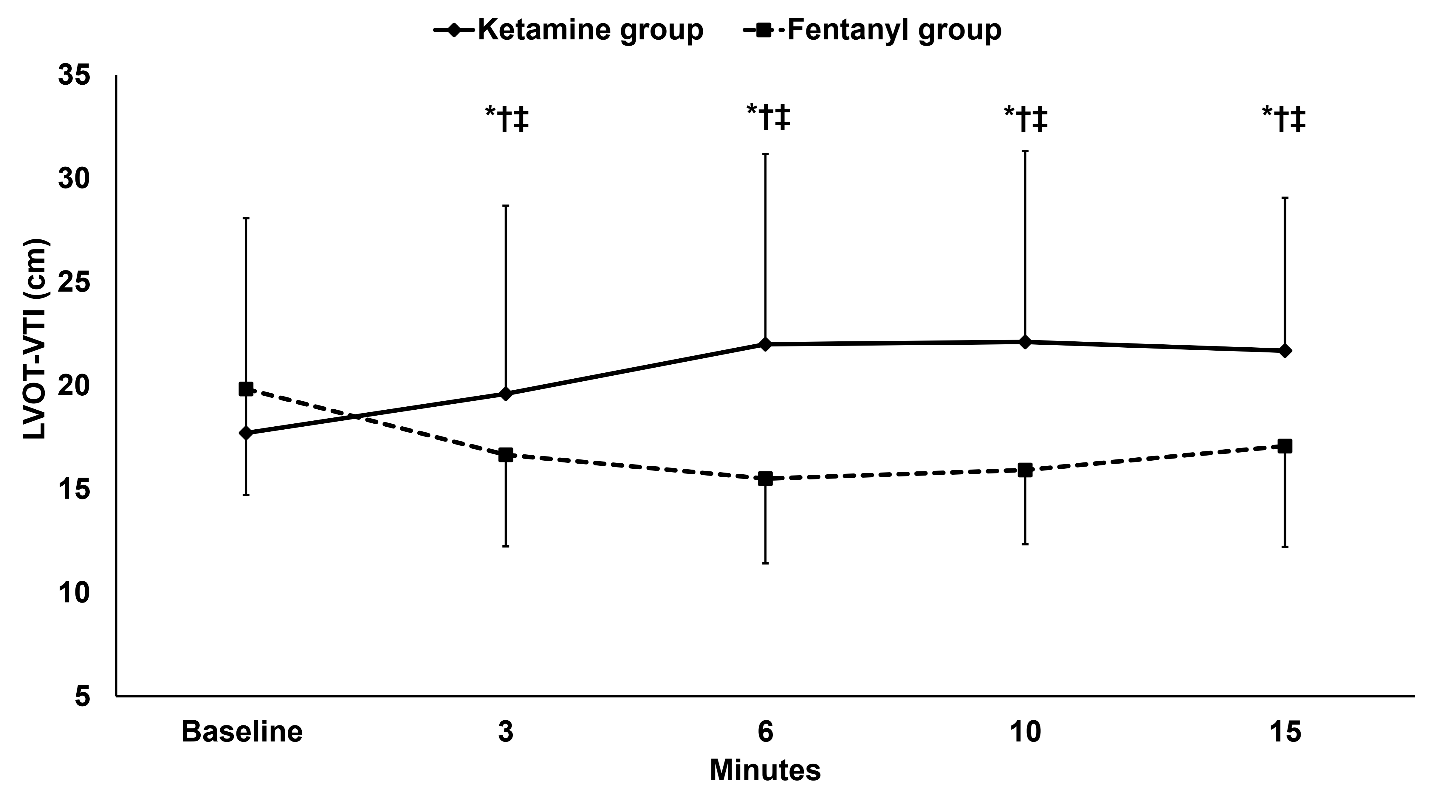


Supplementary figure 1: LVOT-VTI. Markers are means and error bars are standard deviations. * denotes statistical significance between both groups, † denotes statistical significance compared to the baseline reading within the ketamine group, ‡ denotes statistical significance compared to the baseline reading within the fentanyl group. LVOT-VTI: left ventricular outflow-velocity time integral


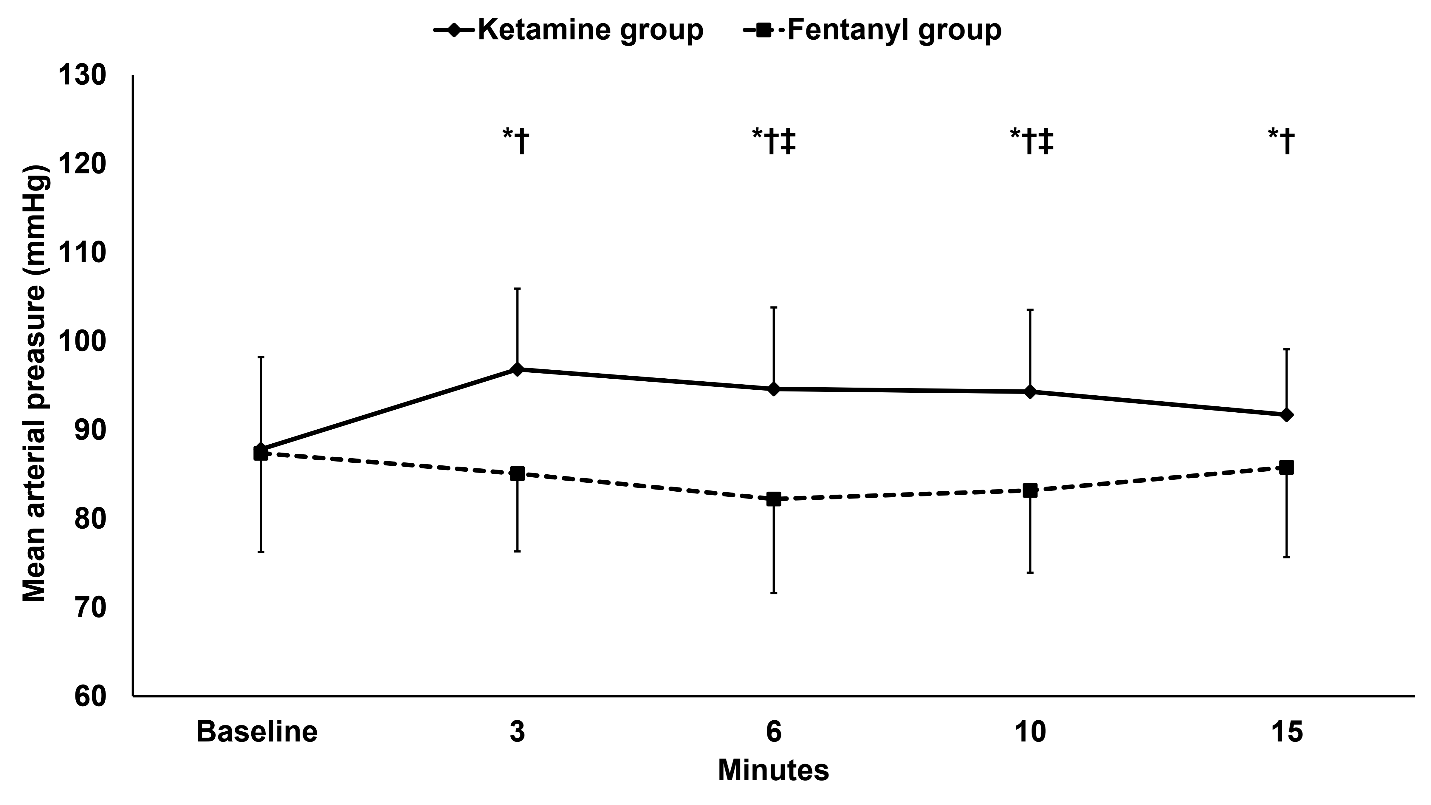


Supplementary figure 2: Mean arterial pressure. Markers are means and error bars are standard deviations. * denotes statistical significance between both groups, † denotes statistical significance compared to the baseline reading within the ketamine group, ‡ denotes statistical significance compared to the baseline reading within the fentanyl group


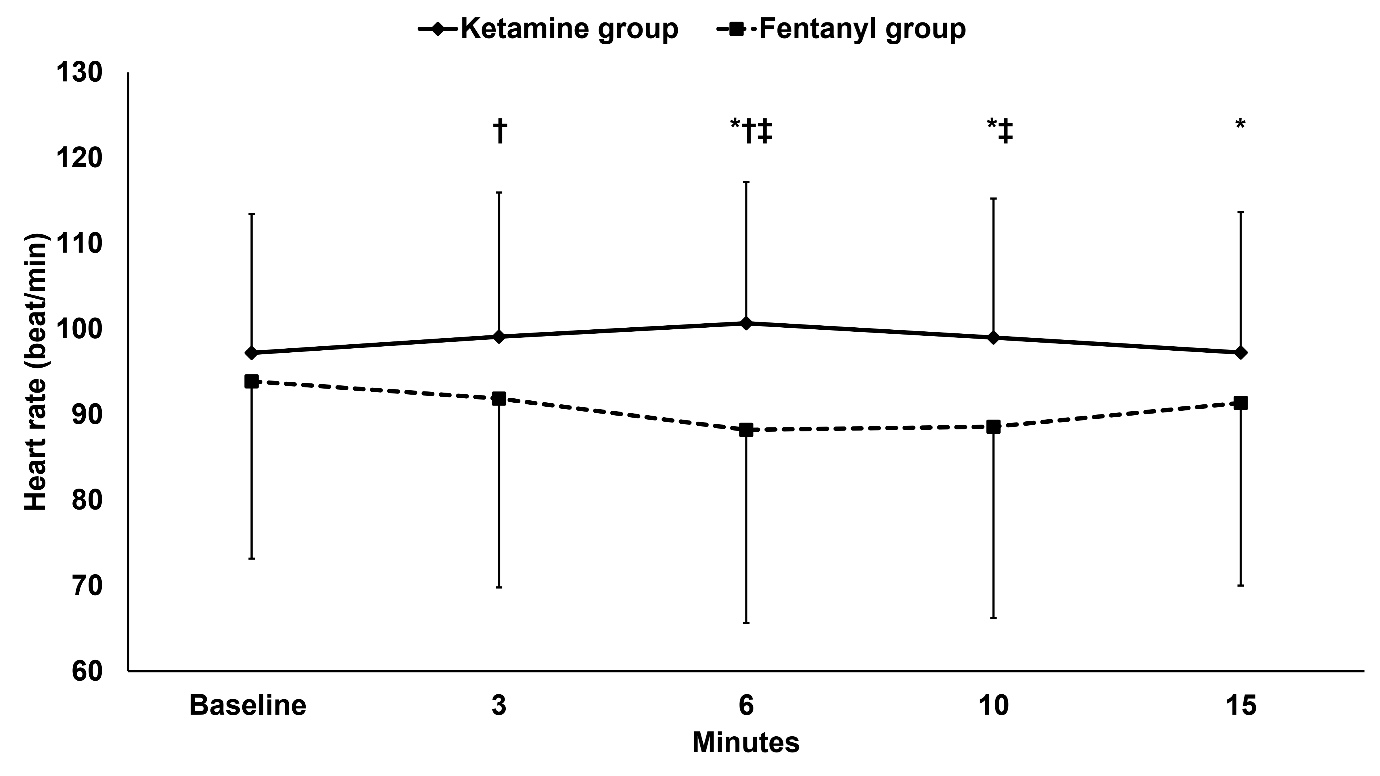


Supplementary figure 3: Heart rate. Markers are means and error bars are standard deviations. * denotes statistical significance between both groups, † denotes statistical significance compared to the baseline reading within the ketamine group, ‡ denotes statistical significance compared to the baseline reading within the fentanyl group
